# PCSK9 inhibitors: a promising lipid-lowering strategy for kidney transplant recipients

**DOI:** 10.1080/0886022X.2025.2604881

**Published:** 2026-01-01

**Authors:** Bohan Luo, Ling Guo, Wenjia Di

**Affiliations:** ^a^ School of Medicine, University of Electronic Science and Technology of China, Chengdu, China; ^b^ Organ Transplantation Center, Sichuan Provincial People’s Hospital, University of Electronic Science and Technology of China, Chengdu, Sichuan, China

**Keywords:** PCSK9 inhibitors, kidney transplantation, lipid management, clinical applications, research progress

## Abstract

Recent advancements in transplant techniques and immunosuppressive drugs have improved long-term survival rates and increased the age of transplant recipients. Dyslipidemia has become a major complication, leading to atherosclerotic cardiovascular disease as the primary cause of renal function loss and mortality, surpassing rejection. Although statins are the first-line treatment, many patients face limitations due to drug interactions, side effects, and renal function impacts, resulting in suboptimal lipid control and limited clinical benefits. Thus, there is a clear need for alternative lipid-lowering treatments for kidney transplant recipients. Proprotein convertase subtilisin/kexin type 9 (PCSK9)inhibitors have shown promise in cardiovascular disease management in the general population, but research on their use in kidney transplant patients is limited to case reports and small-scale studies. This article reviews the progress of PCSK9 inhibitors in lipid management for these patients, discussing their mechanisms, metabolism, clinical applications, efficacy, and safety, while providing insights for future research and clinical practice to enhance blood lipid management guidelines and improve the quality of life and survival rates of kidney transplant recipients.

## 1. Introduction

**Figure 1.**
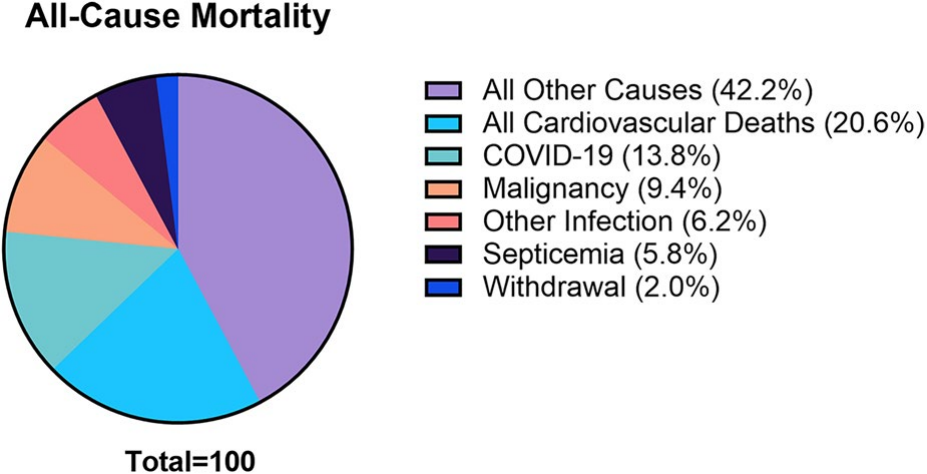
Composition ratio of all-cause mortality in patients after kidney transplantation (without inclusion of missing and unknown causes of death in patients with kidney transplant who died in 2022).

Kidney transplantation is an important means of saving the lives of patients with end-stage renal disease and can significantly improve patient prognosis. However, the metabolic challenges that follow, particularly post-operative dyslipidemia, have become a primary threat to the long-term quality of life and cardiovascular health of these patients. Current research indicates that the prevalence of dyslipidemia after kidney transplantation is as high as 80% [1]. According to data from the US Renal Data System in 2024, 32% of patients die from cardiovascular disease, and all cardiovascular events account for 20.6% of all-cause mortality, making them the leading cause of death among all known causes of death [2]. Hyperlipidemia is the main risk factor of atherosclerotic cardiovascular disease (ASCVD), so dyslipidemia is bound to be closely related to the incidence of cardiovascular events [3,4]. It is important to acknowledge that kidney transplant recipients are inherently exposed to multiple non-modifiable risk factors, including advanced age, baseline function of the allograft, preoperative dialysis vintage, and pre-transplant hyperphosphatemia. Nevertheless, a notable opportunity for intervention exists: postoperative dyslipidemia in these patients can still be substantially ameliorated through the implementation of proactive and individualized lipid management strategies. Therefore, effective monitoring and intervention of dyslipidemia in kidney transplant recipients is a fundamental approach to reducing the occurrence of cardiovascular events. The all-cause mortality of patients after kidney transplantation is shown in Figure 1.

Statins are the primary treatment for dyslipidemia in kidney disease patients, but their use in transplant recipients is limited due to interactions with immunosuppressants, potential liver and kidney function impacts, and side effects like muscle pain and diabetes, making it challenging to achieve ideal lipid levels of patients [5,6]. Thus, new lipid-lowering strategies are urgently needed for these patients. Proprotein convertase subtilisin/kexin type 9 (PCSK9) inhibitors are a new class of lipid-lowering drugs that effectively reduce low-density lipoprotein cholesterol (LDL-C) levels by inhibiting the function of the PCSK9 protein, thereby increasing the number of low-density lipoprotein receptors (LDLR) on the liver surface. In recent years, research on PCSK9 inhibitors for the prevention of cardiovascular disease in the general population has been widely conducted, showing good efficacy and safety. However, the application of PCSK9 inhibitors in kidney transplant recipients is still in the exploratory stage, and related studies are very scarce. Therefore, exploring the potential value of PCSK9 inhibitors in treating dyslipidemia in kidney transplant recipients and conducting drug-related clinical research is of significant clinical importance.

The high incidence of dyslipidemia after kidney transplantation is associated with multiple contributing factors. First, the metabolic effects of immunosuppressive agents play a significant role: calcineurin inhibitors (such as cyclosporine A and tacrolimus) can increase serum total cholesterol, LDL-C, and triglycerides by downregulating LDLR expression and promoting fatty acid synthesis, with cyclosporine A exhibiting more pronounced effects. Glucocorticoids stimulate lipolysis and inhibit fatty acid re-synthesis, leading to elevated plasma free fatty acids and subsequent conversion into triglycerides; long-term use may also induce obesity and insulin resistance, further exacerbating lipid abnormalities. Mammalian target of rapamycin (mTOR) inhibitors (such as sirolimus), while reducing fatty acid synthesis *via* inhibition of fatty acid synthase, may interfere with insulin signaling pathways, increasing the risk of post-transplant insulin resistance and hyperglycemia, thereby indirectly affecting lipid metabolism. Mycophenolate mofetil has a relatively mild impact on lipid metabolism, yet combination therapy can still aggravate dyslipidemia [7,8]. Secondly, alterations in the body’s metabolic state post-transplantation represent significant contributing factors. Although preexisting dyslipidemia from the end-stage renal disease phase may be partially ameliorated by transplantation, impaired allograft function or drug-induced metabolic stress can hinder complete normalization of the lipid profile. The high incidence of post-transplant proteinuria, insulin resistance, and new-onset diabetes mellitus accelerates atherogenesis by enhancing very-low-density lipoprotein (VLDL) synthesis in the liver and reducing high-density lipoprotein cholesterol (HDL-C) [9]. Furthermore, postoperative weight gain, particularly central obesity, nutritional imbalances, and metabolic syndrome contribute to dyslipidemia through multiple pathways [10]. Lifestyle factors also play a non-negligible role: diets high in saturated and trans fats directly elevate serum triglycerides and LDL-C while lowering HDL-C. Physical inactivity following surgery reduces lipid turnover and exacerbates adipose accumulation. Smoking, alcohol consumption, chronic sleep disturbances, and psychological stress further deteriorate lipid profiles and damage vascular endothelium by activating inflammatory pathways and interfering with lipid-metabolizing enzyme activities [11–13].

In summary, post-operative lipid management in kidney transplant recipients is a complex and important clinical issue. The limitations of traditional statin therapy are no longer sufficient to address the current challenges, while PCSK9 inhibitors, as an emerging treatment option, may provide new solutions to improve this situation. The following sections of this article will discuss in detail the potential of PCSK9 inhibitors to replace statins or be used in combination with statins as a first-line recommended treatment in the kidney transplant recipient population.

**Figure 2.**
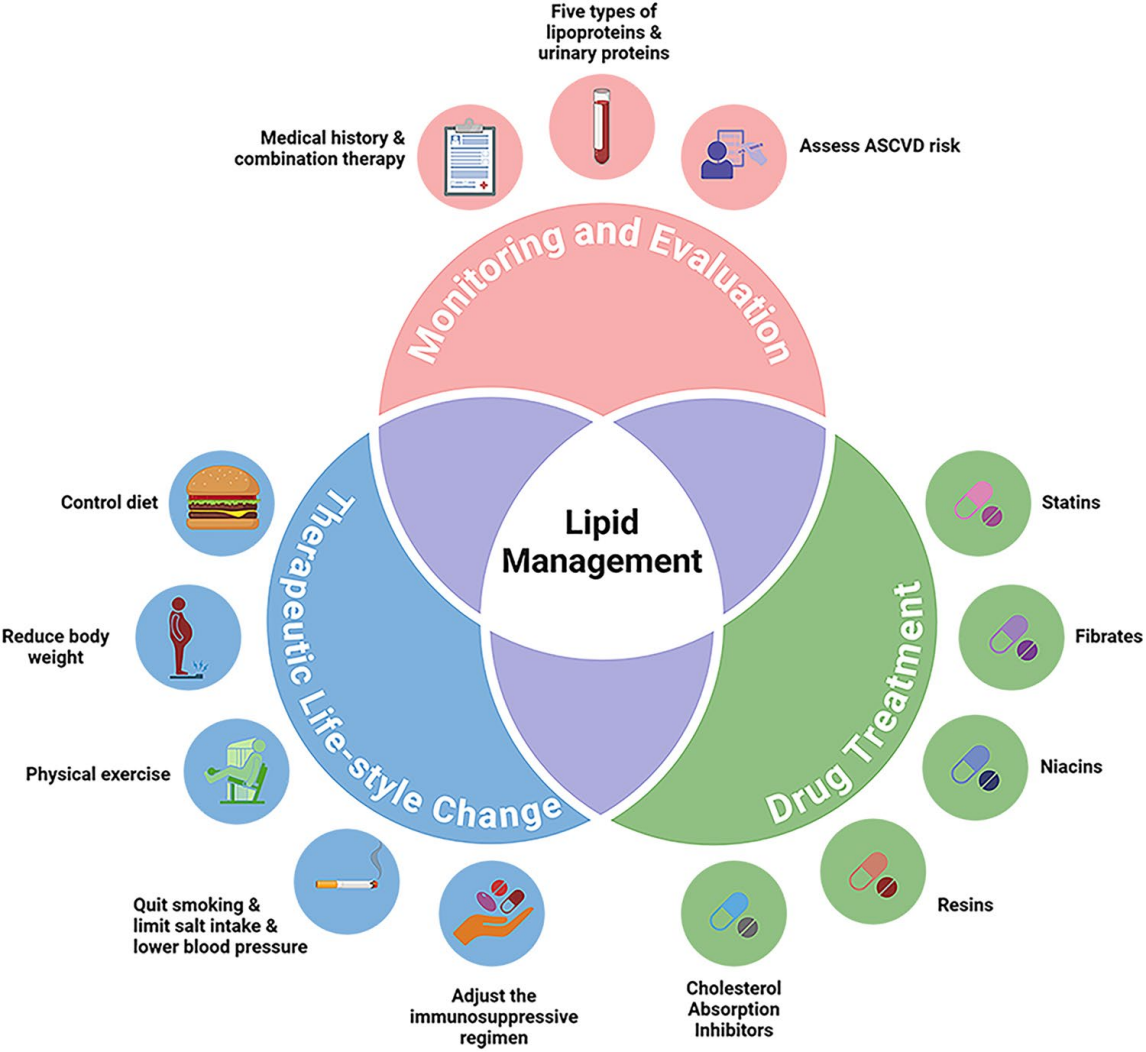
Lipid management in kidney transplant recipients.

The current blood lipid management plan for patients after kidney transplantation is shown in Figure 2.

## 2. Materials and methods

### 2.1. Literature retrieval strategy

This review systematically searched PubMed, Embase, the Cochrane Library, China National Knowledge Infrastructure, and Wanfang Data Knowledge Service Platform from database inception to October 2024, using a combination of search terms including ‘PCSK9 inhibitors’, ‘proprotein convertase subtilisin/kexin type 9 inhibitors’, ‘kidney transplantation’, ‘renal transplant recipients’, ‘dyslipidemia’, and ‘lipid management’. Chinese and English search terms were adapted according to the specific database requirements. Additionally, the reference lists of included publications were manually screened to identify any additional relevant studies.

### 2.2. Literature inclusion and exclusion criteria

Inclusion criteria: (1) Study population comprising kidney transplant recipients; (2) Research content involving the mechanism, efficacy, safety, or clinical application of PCSK9 inhibitors; (3) Study types including randomized controlled trials (RCTs), cohort studies, case reports, basic experimental research, and published reviews; (4) Full-text availability in either English or Chinese, with clearly extractable data. Exclusion criteria: (1) Studies on PCSK9 inhibitors in non-kidney transplant populations; (2) Conference abstracts, dissertations, and unpublished grey literature; (3) Duplicate publications or studies with low quality scores (JBI quality assessment tool score < 3).

### 2.3. Literature screening and data extraction

Literature screening and data extraction were independently conducted by two researchers with professional medical backgrounds to ensure rigor and accuracy. The screening process began with a thorough review of titles and abstracts to promptly exclude publications that clearly did not meet the inclusion criteria. During this initial phase, studies were excluded based on predefined inclusion and exclusion criteria, such as irrelevant research topics, non-conforming study populations, or unsuitable methodologies. Subsequently, potentially relevant articles that passed the initial screening underwent full-text review. In this stage, the inclusion and exclusion criteria were reapplied to finalize the selection of eligible studies. For data extraction, a standardized form was developed to collect key information, including: Study design: specifying the type of research (e.g., prospective, retrospective, randomized controlled trial); Sample size: accurately recording the number of participants enrolled in each study; Intervention details: describing the types of PCSK9 inhibitors (e.g., evolocumab, alirocumab), dosage, and administration protocols (including frequency and duration of treatment); Outcome measures: focusing on lipid profile changes (e.g., LDL-C, HDL-C, triglycerides), cardiovascular events (e.g., incidence of myocardial infarction, stroke, cardiovascular death), and adverse effects (e.g., injection site reactions, flu-like symptoms, elevated liver enzymes); Key conclusions: summarizing the authors’ interpretations and findings.

In case of disagreement between the two researchers during the literature screening or data extraction process, a third senior researcher was consulted to participate in the discussion. Through thorough communication and analysis, a consensus was reached based on the study criteria and professional expertise. After rigorous screening and extraction, a total of 108 articles, all in English, were ultimately included in this review. These studies provide a solid data foundation and theoretical support for the subsequent in-depth analysis of the application of PCSK9 inhibitors in the relevant field.

## 3. Synthesis of evidence on PCSK9 inhibitors

### 3.1. Structure and function of PCSK9

The PCSK9 gene is located on human chromosome 1 and contains 12 exons and 11 introns [14]. PCSK9 is a protein with multiple functional domains, including an N-terminal proregion and a C-terminal catalytic region, which is primarily composed of a catalytic triad of histidine, aspartic acid, and serine that is crucial for maintaining enzymatic activity. Additionally, PCSK9 has a C-terminal region rich in histidine and cysteine, which is believed to play an important role in binding to LDLR. Studies have found that this region forms a stable complex when PCSK9 binds to LDLR, thereby mediating the endocytosis and degradation of LDLR [15,16], which reduces the number of LDLRs on the cell surface and leads to elevated plasma LDL cholesterol levels [17,18]. The expression of PCSK9 is regulated by various factors, including cholesterol levels and the activation of the transcription factor sterol regulatory element-binding protein 2 (SREBP2) [19,20]. The concentration of cholesterol directly affects the transcription level of PCSK9, thereby influencing the number of LDLRs. For example, high cholesterol levels stimulate the expression of PCSK9, leading to the degradation of LDLR and an increase in cholesterol levels in plasma, a mechanism that is particularly evident in familial hypercholesterolemia (FH). There is a close relationship between the structure and function of PCSK9; certain mutations in PCSK9 can lead to structural changes. For instance, some pathogenic mutations (such as p.E32K and p.R469W) can cause conformational changes in PCSK9, thereby affecting its ability to degrade LDLR, a mechanism that is also closely related to the occurrence of familial hypercholesterolemia [21]. Furthermore, the relationship between the function and structure of PCSK9 provides an important theoretical basis for the design of PCSK9 inhibitors. By targeting these structural features, it is possible to effectively intervene in its interaction with LDLR, thereby lowering plasma cholesterol levels and reducing the incidence of cardiovascular events [22].

### 3.2. The role of PCSK9 in lipid metabolism

PCSK9 not only plays an important role in the regulation of LDL receptors but also has a crucial role in cholesterol metabolism. Studies have shown that PCSK9 synthesized in the liver has a regulatory effect on cholesterol homeostasis, affecting cholesterol uptake and excretion. The expression of PCSK9 is positively correlated with liver cholesterol content and negatively correlated with bile acid content, indicating that PCSK9 may participate in the regulation of cholesterol metabolism by influencing the process of converting cholesterol into bile acids [23]. Furthermore, inhibition of PCSK9 can promote the nuclear expression of PPARα, thereby activating the transcription of CYP7A1 and enhancing the ability to convert cholesterol into bile acids, which lowers cholesterol levels and prevents the formation of cholesterol stones. Another study revealed that PCSK9 regulates the body’s lipid metabolism through complex interactions with the gut microbiota. Research indicates that the expression of PCSK9 may be influenced by the gut microbiota, and conversely, PCSK9 may also affect the composition and function of the microbiota by regulating the host’s metabolic environment. For example, certain microbes can regulate the expression of PCSK9 by producing metabolites such as short-chain fatty acids, thereby affecting cholesterol metabolism and absorption. Additionally, dysbiosis of the gut microbiota may lead to abnormal increases in PCSK9 levels, exacerbating lipid metabolic disorders and the occurrence of related diseases [24].

### 3.3. Classification and mechanism of action of PCSK9 inhibitors

#### 3.3.1. Comparison of different types of PCSK9 inhibitors

As we gain a deeper understanding of PCSK9 function, inhibitors targeting it have gradually become important drugs for treating hypercholesterolemia. PCSK9 inhibitors started being developed in 2003, and with a series of successful clinical trials, these drugs have shown significant ability to lower LDL-C levels and have also achieved positive results in reducing cardiovascular events. Currently, several PCSK9 inhibitors have been approved for market, categorized into first, second, and third generations (Table 1), showing good safety and efficacy in clinical applications [25].

**Table 1.** Summary of relevant agents.

Name	Year of approval	Type	Key markets	Approximate monthly cost	Clinical trial status
Evolocumab	2015	First-generation PCSK9 inhibitor	America	$ 112.32–$ 182.52	Approved
Alirocumab	2015	First-generation PCSK9 inhibitor	America	$ 41.42–$ 78.62	Approved
Inclisiran	2020	Second-generation PCSK9 inhibitor	Europe	$ 1,402.32	Approved
Tafolecimab	2023	First-generation PCSK9 inhibitor	China	$ 42.12	Approved
Recaticimab	2025	Third-generation PCSK9 inhibitor	China	$ 233.06	Approved

The first generation of PCSK9 inhibitors includes monoclonal antibodies (mAbs) represented by evolocumab, alirocumab, and tafolecimab. Monoclonal antibodies are antibodies that target specific antigens, with high specificity and affinity.

The development of evolocumab commenced in 2006, initially aiming to address suboptimal responses to conventional therapies, such as statins, in certain hypercholesterolemia patients. The discovery of PCSK9 provided a critical target for novel cholesterol-lowering agents. In 2015, evolocumab received approval from the U.S. Food and Drug Administration (FDA), becoming one of the first PCSK9 inhibitors to enter the market. Clinical trial results demonstrated its excellent efficacy in reducing LDL-C levels. When combined with statins, it significantly further lowered LDL-C and showed favorable prognostic benefits in reducing cardiovascular event risks. For instance, in the ODYSSEY OUTCOMES trial, the evolocumab group exhibited a significantly lower incidence of cardiovascular events compared to the control group [26]. Furthermore, the long-term safety profile of evolocumab has been established, with studies indicating a low incidence of adverse effects, primarily including injection site reactions and allergic responses [27]. It should be noted that, as a first-generation PCSK9 inhibitor, evolocumab may rarely induce non-immunoglobulin E (IgE)-dependent T-cell–mediated cytokine reactions. Although such cases are uncommon and should not be overemphasized, they warrant attention in clinical practice. The primary indication for evolocumab is the treatment of hypercholesterolemia, particularly in patients with inadequate response to or intolerance of statins. Additionally, it is indicated for secondary prevention in individuals at high cardiovascular risk. Liver function should be monitored during treatment, as PCSK9 is synthesized in the liver and hepatic impairment may affect drug metabolism [28]. Regular monitoring of cholesterol levels and liver function is recommended throughout therapy to ensure safe and effective use.

Alirocumab has a half-life of approximately 17–20 days, allowing for dosing every 2 weeks or monthly, with good patient tolerance and sustained efficacy [29]. The drug exhibits minimal interindividual variability in bioavailability, is primarily metabolized in the liver, and is cleared *via* cell-mediated endocytosis [30]. Additional studies indicated that alirocumab can reduce LDL-C levels by up to 62% in high cardiovascular risk patients [31]. Common adverse effects include injection site reactions, flu-like symptoms, and allergic responses [32]. In large-scale ODYSSEY trials, its overall incidence of adverse events was similar to that of the placebo group [33]. However, severe allergic reactions (e.g., anaphylaxis), though extremely rare, may occur in a small number of patients [34]. Notably, as a first-generation PCSK9 inhibitor, case reports suggest that alirocumab may rarely induce non-IgE-dependent T-cell-mediated cytokine reactions. Although the probability of such events is low, clinicians should remain vigilant for related abnormal reactions, such as unexplained fever or worsening rash, during clinical application to ensure timely intervention.

Tafolecimab is a novel first-generation PCSK9 inhibitor with a mechanism of action similar to that of alirocumab and evolocumab, yet it exhibits higher specificity and a lower potential for eliciting immune reactions. Pharmacokinetic studies indicate that tafolecimab has a longer *in vivo* half-life, which allows for reduced dosing frequency and may improve patient adherence [35]. Furthermore, clinical trials have demonstrated that tafolecimab effectively lowers LDL-C levels with consistent efficacy across diverse populations, along with a favorable safety and tolerability profile. Although tafolecimab is well-tolerated, potential immunogenic reactions should be monitored during long-term use. In addition, based on clinical observations of other first-generation PCSK9 inhibitors, while reported cases of non-IgE-dependent T-cell-mediated cytokine reactions associated with tafolecimab remain rare, vigilance is warranted in clinical practice due to the class-related risk. Close monitoring for immune-related adverse events following administration is essential to ensure treatment safety.

#### 3.3.2. Second-generation PCSK9 inhibitor

The second generation of PCSK9 inhibitors consists of small interfering RNA (siRNA), with inclisiran as the representative drug.

siRNA is an important gene regulatory molecule that utilizes the RNA interference (RNAi) mechanism to inhibit gene expression by specifically targeting and degrading specific messenger RNA (mRNA). siRNA is typically generated from long double-stranded RNA (dsRNA) through cleavage by the Dicer enzyme, forming short RNA with specific sequences. One strand of siRNA is loaded into the RNA-induced silencing complex, which then binds to the target mRNA, leading to its degradation and reducing the synthesis of the target protein. Based on this mechanism, researchers began exploring the use of siRNA to inhibit PCSK9 expression. Inclisiran, as a double-stranded siRNA, is designed to target PCSK9 mRNA, thereby reducing its protein synthesis and lowering LDL cholesterol levels [36]. Inclisiran’s clinical development has gone through several phases, with the ORION clinical trial series being the most important. These trials evaluated the safety and efficacy of inclisiran in different populations, including high-risk cardiovascular disease patients and hypercholesterolemia patients. Key trials such as ORION-9, ORION-10, and ORION-11 showed that inclisiran could significantly lower LDL cholesterol levels and exhibited good tolerance during use. Specifically, inclisiran can reduce LDL cholesterol by approximately 50% in patients receiving the maximum tolerated dose of statins [37]. Studies in different populations indicate that inclisiran has consistent efficacy in lowering LDL-C. For instance, in patients with ASCVD, inclisiran’s LDL-C reduction reached 51.5%, showing good effects across different subgroups (such as age, sex, race) [38]. For patients with diabetes or chronic kidney disease, inclisiran also demonstrated significant LDL-C lowering effects, with good safety, where injection site reactions were the most common side effects, typically mild [39]. In high-risk patients, the combination of inclisiran with statins showed good synergistic effects. Research indicates that inclisiran, as an adjunct therapy to statins, can significantly enhance LDL-C reduction. For example, in the ORION-10 and ORION-11 trials, the reduction in LDL-C with the combination of inclisiran and statins was significantly greater than that in patients using statins alone. In 2021, inclisiran was approved by the FDA and the European Medicines Agency (EMA), becoming the first siRNA drug for treating hypercholesterolemia [40]. The administration of inclisiran is done through subcutaneous injection every 6 months, greatly improving patient compliance, especially for those who struggle to reach cholesterol targets with traditional treatments, providing a new treatment option [41]. Despite inclisiran showing good effects in clinical applications, the potential risks of long-term use are not yet fully understood.

#### 3.3.3. Third-generation PCSK9 inhibitor

The third-generation PCSK9 inhibitors are represented by ultra-long-acting PCSK9 monoclonal antibodies such as recaticimab, and also include novel formulations like the oral cyclic peptide MK-0616 and the fusion protein lerodalcibep.

The development of recaticimab aims to address the high dosing frequency associated with traditional PCSK9 inhibitors. Early studies have demonstrated its significant efficacy in reducing LDL-C levels in healthy volunteers. Notably, it further lowers LDL-C in patients already on stable statin therapy. In a randomized, double-blind, placebo-controlled phase I/II clinical trial, recaticimab was evaluated for safety and efficacy. Results showed substantial LDL-C reduction across various doses and dosing intervals, with no serious adverse events reported [42]. The clinical development of recaticimab includes multiple phases, with phase III trials being particularly critical. In the REMAIN-1 study, a randomized, double-blind, placebo-controlled trial, 703 patients were randomized to receive either recaticimab at different doses or placebo. The results indicated LDL-C reductions of 49.6%, 52.8%, and 45.0% with dosing regimens of 150 mg every 4 weeks, 300 mg every 8 weeks, and 450 mg every 12 weeks, respectively [43]. Compared to other PCSK9 inhibitors, recaticimab demonstrates comparable or superior efficacy with a more flexible dosing schedule. Its extended dosing intervals may improve patient adherence relative to currently approved agents such as alirocumab and evolocumab. Studies indicate that recaticimab achieves comparable LDL-C reduction while reducing the frequency of medical visits and medication burden, which is particularly important for high-risk patients requiring long-term therapy [44]. Furthermore, the safety profile of recaticimab is similar to that of placebo, with treatment-related adverse events occurring in 28.5% of the recaticimab group versus 26.6% in the placebo group [45]. Evaluation across diverse patient populations shows that recaticimab effectively lowers LDL-C in various high-risk groups. It exhibits particularly pronounced efficacy in reducing LDL-C in patients with a history of ASCVD. Additionally, recaticimab demonstrates favorable efficacy as an adjunctive therapy in patients receiving statin treatment [46]. Moreover, it shows consistent therapeutic effects across different age groups and both sexes [47]. MK-0616, an oral cyclic peptide PCSK9 inhibitor, offers distinct advantages. Its oral administration significantly enhances patient convenience and adherence, addressing the limitations of injectable PCSK9 inhibitors in terms of usability. In preclinical studies and early clinical trials, MK-0616 has demonstrated potent LDL-C-lowering efficacy by specifically binding to PCSK9 and inhibiting its interaction with the LDLR, thereby promoting LDL-C clearance [48,49]. Ongoing research continues to evaluate its efficacy and safety in diverse populations, including special groups such as kidney transplant recipients, and to explore its optimal application in lipid management. The fusion protein lerodalcibep functions through an innovative molecular design, combining the PCSK9-binding domain with an immunoglobulin constant region to achieve high affinity and an extended half-life. Clinical studies noted that lerodalcibep effectively reduces LDL-C levels with less frequent dosing due to its prolonged dosing interval. In trials involving patients with hypercholesterolemia, lerodalcibep exhibited a favorable tolerability profile, with fewer and milder common adverse events [50]. For kidney transplant recipients, who require long-term lipid management and often present with multiple comorbidities, the prolonged efficacy and favorable safety of lerodalcibep may offer a potential treatment option. However, dedicated studies in this specific population are still needed to clarify its clinical utility.

Beyond their lipid-lowering effects, PCSK9 inhibitors have demonstrated broader clinical value, as evidenced by numerous basic and clinical studies in recent years. These agents show potential benefits in improving lipid profiles, modulating coagulation function, attenuating transplant rejection, and preserving renal function, thereby providing a stronger theoretical foundation for their application in special populations such as kidney transplant recipients.

In conventional lipid-lowering therapy, elevating HDL levels has been challenging to achieve with single-agent regimens. PCSK9 inhibitors demonstrate unique potential in this regard. Giugliano et al. conducted a randomized double-blind study focusing on PCSK9 inhibitors, enrolling 27,564 patients with established atherosclerosis who were receiving statin therapy. The results indicated that, compared with statin monotherapy, the addition of a PCSK9 inhibitor not only further reduced LDL-C levels but also increased HDL levels, thereby enhancing the anti-atherogenic role of HDL [51]. Research by Jin et al. on the mechanism by which homocysteine (Hcy) accelerates atherosclerosis further revealed a fundamental link between PCSK9 and HDL function. In *in vitro* experiments using THP-1 macrophages, Hcy up-regulated PCSK9 gene and protein expression in a dose- and time-dependent manner. PCSK9 was found to inhibit ATP-binding cassette transporter A1 and G1 (ABCA1, ABCG1)-mediated cholesterol efflux, reducing cholesterol transport to apoA-I and HDL, ultimately leading to lipid accumulation in macrophages. In *in vivo* experiments using ApoE^-^/^-^ mice, methionine diet-induced hyperhomocysteinemia exacerbated atherosclerotic lesions in the aortic root. Treatment with the PCSK9 inhibitor SBC-115076 up-regulated ABCA1 and ABCG1 expression in plaque macrophages, restored cholesterol efflux capacity, reduced lipid deposition, and simultaneously lowered plasma Hcy levels while improving the lipid profile, thereby attenuating atherosclerosis severity [52]. Collectively, these studies, spanning both clinical application and mechanistic exploration, demonstrate the significant value of PCSK9 inhibitors in enhancing HDL functionality and attenuating the progression of atherosclerosis, thereby providing a robust theoretical and practical foundation for the prevention and treatment of cardiovascular diseases.

Furthermore, the association between PCSK9 and platelet function has emerged as a key research focus, with growing evidence supporting the value of its inhibitors in the antithrombotic domain [53,54]. At the clinical level, a cross-sectional study by Wang S et al. involving subjects not taking statins or antiplatelet agents provided critical data supporting this association. The study enrolled 89 participants and measured ADP-induced platelet aggregation rate using impedance aggregometry, plasma PCSK9 levels *via* ELISA, and simultaneously assessed serum lipid profiles (total cholesterol, HDL-C, triglycerides, LDL-C). Multiple regression analysis demonstrated that, after adjusting for potential confounders including total cholesterol, LDL-C, platelet count, gender, and smoking status, PCSK9 remained an independent predictor of ADP-induced maximum platelet aggregation rate. This confirms an independent positive correlation between PCSK9 and platelet reactivity, particularly highlighting its regulatory role in ADP-mediated platelet activation pathways [55]. Moreover, the study by Ziogos et al. involving patients with acute coronary syndrome (ACS) further expanded the understanding of PCSK9’s impact on the cardiovascular system. In this study, 420 ACS patients were randomized within 24 h of hospitalization to receive either a single 90 mg dose of evolocumab or placebo. Levels of platelet activation markers (platelet factor 4, P-selectin) and endothelial dysfunction markers (von Willebrand factor) were measured at baseline and at 30 days. The study concluded that PCSK9 inhibition reduced markers of platelet activation and endothelial dysfunction in ACS patients. Additionally, PCSK9 was found to be associated with platelets and vascular endothelial cells in the LIMAsegment, and PCSK9 inhibition reduced this interaction [56]. These studies collectively illustrate the multifaceted role of PCSK9: the former reveals its independent regulatory effect on platelet reactivity in healthy individuals, while the latter demonstrates that its inhibitor reduces markers of platelet activation and endothelial dysfunction in ACS patients and diminishes interactions between PCSK9, platelets, and endothelial cells. These findings provide evidence for the value of PCSK9 inhibitors in antithrombotic therapy and vascular homeostasis improvement, partly elucidating the mechanism by which they reduce the risk of adverse cardiovascular events after ACS. PCSK9 function extends beyond lipid metabolism and thromboregulation, playing a key role in transplant immunology, particularly in heart transplant rejection, where mechanistic insights have opened new directions for clinical application [57]. Zhang et al. [58] demonstrated significantly elevated serum PCSK9 levels in both murine models and human heart transplant rejection patients. Genetic knockout of PCSK9 or its neutralization with antibodies prolonged cardiac allograft survival, reduced inflammatory cell infiltration within grafts, and attenuated splenic alloreactive T-cell expansion. Further mechanistic studies revealed that during heart transplant rejection, PCSK9 is primarily secreted by the recipient liver with markedly upregulated expression, accompanied by aberrant activation of tumor necrosis factor-α (TNF-α) and interferon-γ (IFN-γ) signaling pathways, as well as dysregulation of bile acid and fatty acid metabolic pathways. TNF-α and IFN-γ were found to synergistically enhance PCSK9 expression in hepatocytes *via* the transcription factor SREBP2. Moreover, PCSK9 promotes heart transplant rejection) by suppressing CD36 expression on macrophages, reducing fatty acid uptake, and driving macrophages toward a pro-inflammatory phenotype. This enhances their capacity to stimulate donor-reactive T-cell proliferation and IFN-γ production. Experiments using hepatocyte-specific Pcsk9 knockout mice and CD36 pathway validation confirmed that the pro-rejection effect of PCSK9 depends on the recipient’s CD36 pathway. This study is the first to reveal an immune mechanism by which the liver modulates heart transplant rejection through the PCSK9/CD36 axis, expanding the understanding of PCSK9’s role in immunoregulation and offering a novel therapeutic target for preventing transplant rejection. Together with previous findings on PCSK9’s benefits in lipid modification, antithrombotic effects, and vascular endothelial protection, these results underscore its pleiotropic value and provide a theoretical basis for the cross-border application of PCSK9 inhibitors in both cardiovascular and transplant medicine.

Proteinuria is a significant marker of long-term renal injury in kidney transplant recipients, and the potential of PCSK9 inhibitors in reducing proteinuria has been preliminarily explored. A cross-sectional study by Doiron et al. involving 168 patients with nephrotic syndrome demonstrated that plasma PCSK9 concentrations increased with the severity of proteinuria. After adjusting for confounders, the urine protein-to-creatinine ratio remained significantly associated with PCSK9 levels (β = 0.205, *p* = 0.006), suggesting that PCSK9 may be involved in proteinuria-related pathological processes and providing a theoretical basis for further investigation into PCSK9 inhibitors in proteinuria management among transplant recipients [59]. Hummelgaard et al. proposed that PCSK9 exacerbates proteinuria by interacting with and downregulating megalin, a key reabsorption receptor in the proximal tubule. Experimental models showed that inhibiting PCSK9 maintained megalin expression, reduced proteinuria, and improved disease phenotypes. Additionally, PCSK9 inhibitors (monoclonal antibodies and siRNA) were confirmed to effectively lower lipids and exhibit a favorable safety profile in patients with mild to moderate chronic kidney disease [60]. Collectively, these studies expand the understanding of the pleiotropic effects of PCSK9, demonstrating its clinical potential across cardiovascular diseases, organ transplantation, and renal protection. For kidney transplant recipients, particularly those with diabetes or chronic allograft nephropathy, PCSK9 inhibitors may not only lower lipids but also reduce proteinuria and preserve graft function. However, their efficacy and safety in this population require validation through large-scale prospective cohort studies.

#### 3.3.4. The mechanism of action of PCSK9 inhibitors

**Figure 3.**
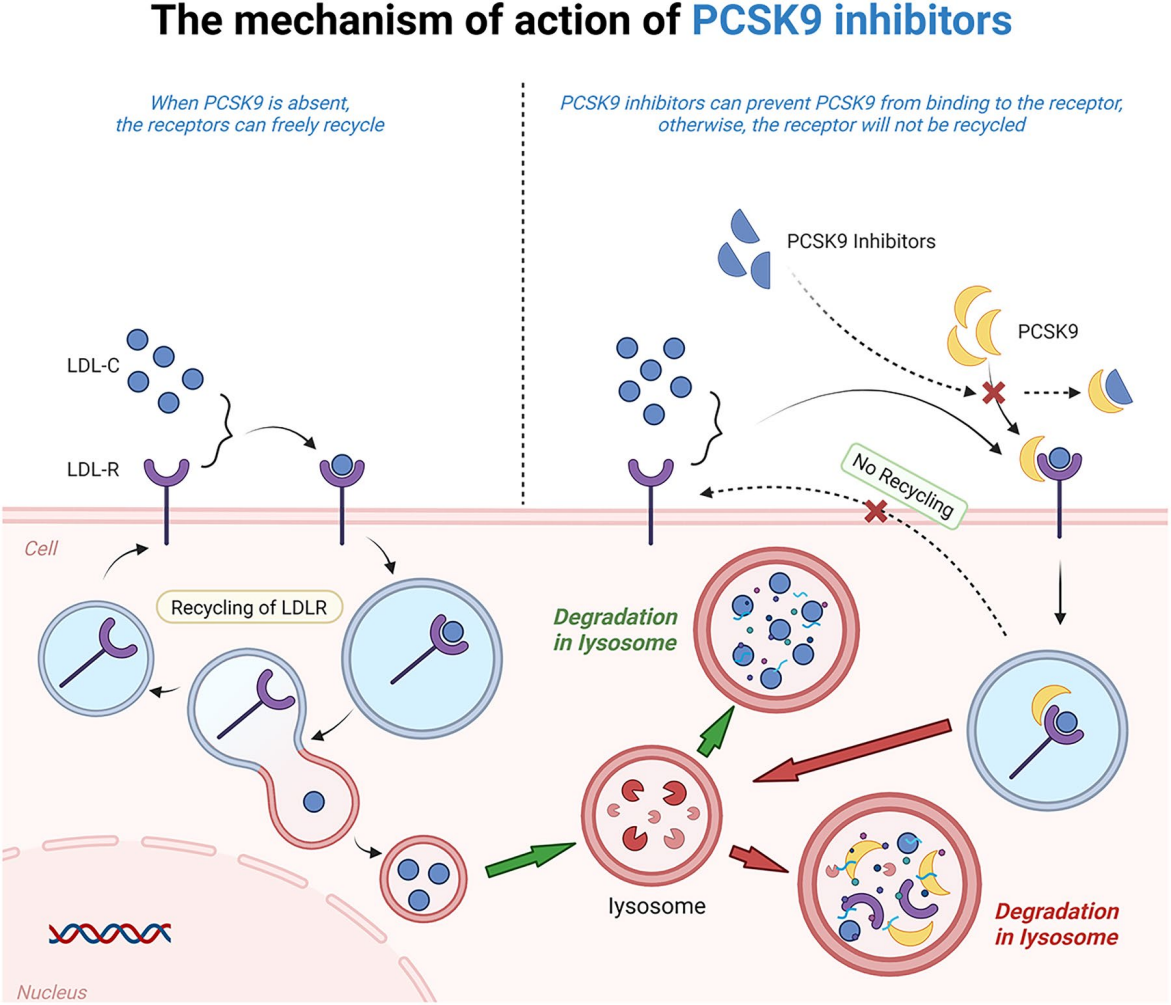
The mechanism of action of PCSK9 inhibitors.

As mentioned earlier, PCSK9 promotes the endocytosis and degradation of LDLR by binding to it, leading to a reduction in the surface LDLR of liver cells, which in turn decreases the clearance rate of LDL cholesterol. PCSK9 inhibitors can effectively block this process, increasing the number of LDLRs on the surface of liver cells, promoting the uptake of LDL cholesterol, and lowering plasma LDL cholesterol levels [61]. Additionally, PCSK9 interacts with other receptors such as Toll-like receptors (TLR) and scavenger receptor B, thereby affecting lipoprotein concentrations and thrombosis. These findings suggest that PCSK9 inhibitors not only influence the degradation of LDLR but may also regulate lipid metabolism and inflammatory responses through various pathways, further impacting the progression of cardiovascular diseases. The mechanism of action of PCSK9 inhibitors (evolocumab) is shown in Figure 3.

### 3.4. Clinical efficacy, safety, and limitations of PCSK9 inhibitors

#### 3.4.1. Evidence of curative effect of PCSK9 inhibitors

In recent years, the results of several large clinical trials have shown that PCSK9 inhibitors have significant effects in lowering LDL-C and reducing cardiovascular events (Table 2). The use of PCSK9 inhibitors (such as evolocumab and alirocumab) can reduce LDL-C levels by more than 50%, especially in high-risk patients. For example, the FOURIER trial demonstrated that evolocumab could lower LDL-C by 59% and reduce the risk of major cardiovascular events by 15% to 20%; the ODYSSEY Outcomes trial found that alirocumab reduced LDL-C by 57% while decreasing the incidence of adverse events by 15%. Although the SPIRE-2 trial had limited results due to early termination, it still showed a 21% risk reduction in the primary composite endpoint with bococizumab [62]. This reduction in LDL-C not only improves patients’ lipid levels but also significantly lowers the risk of cardiovascular events. Additionally, several studies have indicated that the application of PCSK9 inhibitors effectively lowers LDL-C across different populations, with particularly pronounced effects in patients receiving high-intensity statin therapy who do not respond well [63]. In addition to their significant impact on LDL-C, PCSK9 inhibitors also have positive effects on other lipid parameters. Research shows that PCSK9 inhibitors can lead to significant decreases in total cholesterol, non-HDL-C, and apolipoprotein B (ApoB) levels, while HDL-C levels may slightly increase. These changes indicate that PCSK9 inhibitors not only effectively lower harmful LDL-C but may also improve the overall lipid profile, thereby reducing the risk of cardiovascular disease [64,65]. Furthermore, PCSK9 inhibitors also have a certain lowering effect on triglyceride levels, further supporting their important role in lipid metabolism regulation [66].

**Table 2.** Recent published clinical studies.

Year	Study type	Safety	Efficacy	Conclusion
2024	Randomized Controlled Trial	YES	YES	PCSK9 inhibitors are safe and effective with statins for hypercholesterolemia post-kidney transplant, and evolocumab reduces cardiovascular events in high-risk patients [75].
2024	Cohort Study	YES	YES	The research results support the use of evolocumab in patients who cannot achieve ideal lipid levels through conventional treatment [74].
2023	Case Report	YES	YES	PCSK9 inhibitors may be considered a pharmacologic option for treating lipid metabolism disorder and reducing low-density lipoprotein cholesterol in transplant recipients [68].
2022	Case Report	YES	YES	PCSK9 inhibitors provide a new possibility for the treatment of lipid metabolism disorders in kidney transplant patients, demonstrating safety and efficacy even in complex situations [71].
2021	Case Report	YES	YES	In kidney transplant patients, the effectiveness of alirocumab in reducing LDL-C levels is similar to other clinical study results, and it has good compatibility with immunosuppressive therapy [72].
2019	Case Report	25% of patients experienced mild and self-limiting side effects.	YES	PCSK9 inhibitors can lower LDL-C by 60% in transplant patients, achieving treatment targets, with minimal effects on immunosuppressive drugs and no reports of organ rejection or new atherosclerotic disease [73].
2018	Case Report	The use of alirocumab may increase the risk of infection in patients.	YES	The increased infection risk from alemtuzumab in kidney transplant patients, especially with everolimus, necessitates careful risk-benefit assessment and regular monitoring of cholesterol and infection status for safety and efficacy [70].

Thus, it is evident that PCSK9 inhibitors not only effectively lower LDL-C but may also reduce the occurrence of cardiovascular events in high-risk patients, demonstrating their potential in the prevention of cardiovascular diseases.

Given the excellent efficacy and safety of PCSK9 inhibitors in the general population, researchers are attempting to address the current challenges in lipid management for kidney transplant patients. Existing clinical trials show that PCSK9 inhibitors can effectively reduce LDL-C levels in kidney transplant recipients and may improve cardiovascular risk. Although research specifically targeting kidney transplant recipients is still limited, existing case reports and small-scale clinical trials indicate that PCSK9 inhibitors demonstrate good tolerance and efficacy in this patient population. For example, one study reported that a 74-year-old male kidney transplant patient experienced a significant reduction in LDL-C levels after using alirocumab, with no serious adverse reactions occurring during treatment, providing preliminary evidence for the application of PCSK9 inhibitors in kidney transplant recipients [67]. Additionally, systematic evaluations have shown that PCSK9 inhibitors also exhibit potential in reducing the risk of cardiovascular events, especially in patients who have poor tolerance or inadequate response to traditional lipid-lowering medications [68]. Furthermore, PCSK9 inhibitors have shown superior effects compared to statins in kidney transplant patients, particularly in reducing total cholesterol and triglycerides. For instance, a network meta-analysis involving 5,768 kidney transplant patients demonstrated that PCSK9 inhibitors significantly reduced LDL-C levels, and when used in combination with statins, they could further enhance lipid-lowering effects [69].

Ordóñez-Fernández et al. initially reported a case of a 45-year-old kidney transplant patient who developed pneumonia after receiving alirocumab treatment. The patient was initially treated with high-dose statins and ezetimibe, but switched to alirocumab due to worsening liver function. After receiving two doses of alirocumab, the patient developed fever and cough, and was ultimately diagnosed with cardiogenic pneumonia and sepsis, requiring admission to the intensive care unit. After 5 days of treatment, the patient was discharged, but the use of alirocumab was temporarily halted. Ninety days after stopping alirocumab, the patient resumed the medication, but subsequently experienced a respiratory infection. Consequently, the patient’s immunosuppressive therapy was changed from everolimus to azathioprine, which led to an improvement in lipid levels and alleviation of respiratory symptoms. Therefore, they recommended that when using alirocumab, patients’ cholesterol levels and infection risks should be regularly assessed, and consideration should be given to pausing the medication after achieving treatment goals [70].

However, in three recent reports, alirocumab has shown good efficacy in lowering cholesterol levels and is safe in immunosuppressed patients [71,72]. In the case report by Warden, B. A. et al. only 25% of patients experienced mild and self-limiting side effects, and no patients discontinued treatment due to side effects [73]. Furthermore, stronger evidence from cohort studies and RCTs supports the effective and safe use of PCSK9 inhibitors in kidney transplant patients. In the prospective cohort study by Amaro, J. et al. kidney transplant patients treated in the hospital from 15 September 2022 to 11 May 2023, were included. The inclusion criteria were patients who had not achieved low-density lipoprotein cholesterol (c-LDL) treatment goals after receiving high-efficacy statins and ezetimibe, or who experienced adverse reactions (such as muscle pain and elevated liver enzymes) from statins. The research team followed up with 13 patients for at least 6 months, comparing total cholesterol, c-LDL, triglycerides, estimated glomerular filtration rate (eGFR), and urinary albumin levels before and after treatment. A total of 13 kidney transplant recipients began receiving evolocumab treatment, and all recipients continued using their previous lipid-lowering medications during treatment. The study found that by the third month after treatment initiation, the recipients’ total cholesterol and low-density lipoprotein cholesterol (c-LDL) levels significantly decreased (*p* = 0.008, *p* = 0.009), and this reduction was maintained after 6 months (*p* < 0.001, *p* = 0.002). Nevertheless, the study did not find changes in triglycerides, eGFR, urinary albumin, or plasma immunosuppressant levels. Additionally, no cases of acute rejection or adverse reactions were reported, and the study found that evolocumab was more effective than alirocumab in reducing cardiovascular events, with stable kidney function, no changes in proteinuria, and no other safety issues reported [74].

Furthermore, in the study by Alotaibi et al. a total of 197 kidney transplant patients were recruited, primarily consisting of Kuwaiti males aged between 50 and 60 years. They were randomly divided into two groups: one group received evolocumab treatment (140 mg every 2 weeks), while the other group maintained statin therapy. The study compared various indicators between patients using evolocumab and those on statins before and after transplantation. The results showed that although the two groups were similar in weight, height, dialysis type, immunosuppressive regimen, and comorbidities before transplantation, the incidence of diabetes was significantly higher in the statin group (*p* = 0.009). After transplantation, although the cholesterol levels in the evolocumab group were significantly higher than those in the statin group at baseline (*p* < 0.001), this difference reversed after 3 months and remained significantly lower than the statin group during follow-up (*p* < 0.05). Additionally, there were no significant differences in clinical outcomes regarding diabetes, kidney function, and cardiovascular events between the two groups after transplantation (*p* > 0.05). While the evolocumab group had a higher incidence of cardiovascular events at baseline (*p* = 0.001), there were no significant differences in clinical outcomes, transplant results, and the incidence of cardiovascular and cerebrovascular complications between the two groups throughout the study (*p* > 0.05), indicating that evolocumab is a safe and effective adjunct therapy to statins [75].

#### 3.4.2. Safety of PCSK9 inhibitors

PCSK9 inhibitors (such as alirocumab and evolocumab) have shown significantly better lipid-lowering effects compared to statins. A systematic review and network meta-analysis demonstrated that PCSK9 inhibitors can reduce LDL-C levels by approximately 50% [76]. This lipid-lowering effect has been validated in multiple studies, particularly in patients who are intolerant to statins, where PCSK9 inhibitors have shown superior results. Additionally, the lipid-lowering effect of PCSK9 inhibitors when used in combination with other lipid-lowering medications (such as ezetimibe) is also very significant, indicating a good synergistic effect of PCSK9 inhibitors in multi-drug combination therapy.

In comparisons of cardiovascular event rates, PCSK9 inhibitors also outperform statins. Multiple studies have shown that PCSK9 inhibitors can effectively reduce the incidence of adverse cardiovascular events, especially in high-risk patients. Furthermore, PCSK9 inhibitors appear to be safer than statins, as studies have shown that their use in combination with statins does not significantly increase the incidence of cardiovascular events and may even reduce the risk of statin-related side effects. Therefore, considering the lipid-lowering effects and the incidence of cardiovascular events, PCSK9 inhibitors may become the preferred treatment option for patients at high risk of cardiovascular disease.

Moreover, PCSK9 inhibitors also show significant advantages in terms of tolerability. Although statins are the cornerstone of lipid-lowering therapy, their common adverse reactions, such as muscle pain, liver function abnormalities, and increased risk of diabetes, often lead to patients discontinuing or reducing their medication, thereby affecting treatment outcomes [77,78]. In contrast, users of PCSK9 inhibitors report fewer adverse reactions, most of which are mild injection site reactions, and do not affect patients’ glucose metabolism [79]. In one study, the use of PCSK9 inhibitors showed good tolerability in high-risk patients, and when used in combination with statins, did not significantly increase the incidence of adverse reactions [80]. Additionally, PCSK9 inhibitors also demonstrate good efficacy and tolerability even in patients who are intolerant to statins, effectively lowering LDL-C levels without increasing the risk of new-onset diabetes.

#### 3.4.3. The main limitation of PCSK9 inhibitors: the cost

Compared to traditional statins, the use of PCSK9 inhibitors, a new type of lipid-lowering medication, is often limited by their high cost. In a study involving Chinese adults, researchers evaluated the cost-effectiveness of different lipid-lowering treatment regimens, including high-dose statin treatment, statin plus ezetimibe, and the combination of statins with PCSK9 inhibitors. The results showed that the incremental cost-effectiveness ratio (ICER)of adding just PCSK9 inhibitors was 68,910 $ per quality − adjusted life year (QALY), while the ICER for adding ezetimibe was 20,242 $ per QALY, which shows that adding ezetimibe is a more cost-effective choice [33].

In the ORION-10 clinical trial in Australia, the study compared inclisiran with statin monotherapy and found that inclisiran could significantly reduce LDL-C levels. However, from a cost-effectiveness standpoint, if its price stays the same, using inclisiran isn’t seen as cost-effective. The study pointed out that if inclisiran’s price dropped by 60%, it might be seen as a cost-effective option [81].

Healthcare policies and drug pricing strategies differ substantially across countries and regions. In some settings, PCSK9 inhibitors are considered cost-effective for specific patient subgroups, while in others, their economic feasibility is questioned. Therefore, despite the promising clinical efficacy of PCSK9 inhibitors, their economic burden, cost-effectiveness profiles, and variations in healthcare policy support remain key areas of ongoing discussion. Additionally, with the continuous advancement of novel drug development and growing market competition, adjustments to PCSK9 inhibitor pricing strategies may occur, which could influence their clinical accessibility and widespread application[82].

## 4. Discussion and future direction

### 4.1. Key findings

Current evidence indicated that elevated lipoprotein(a) [Lp(a)] levels may attenuate the lipid-lowering efficacy of PCSK9 inhibitors. Mechanistically, increased Lp(a) competes with the LDLR for binding sites, occupying the key targets of PCSK9 inhibitors and thereby impeding their ability to promote LDLR recycling and enhance clearance of low LDL-C [83]. This effect is particularly pronounced in patients carrying LDLR mutations, who already exhibit dyslipidemia due to impaired LDLR function, as elevated serum Lp(a) further exacerbates the suboptimal lipid-lowering response to PCSK9 inhibitors, hindering the achievement of lipid control targets [84]. Elevated Lp(a) is relatively common among renal transplant recipients, and some patients may also carry LDLR mutations due to genetic factors. Therefore, the impact of Lp(a) requires particular attention when using PCSK9 inhibitors in this population.

Romagnuolo et al. demonstrated using various hepatocyte models that PCSK9 inhibits the internalization of Lp(a) *via* the LDLR in an LDLR-dependent manner, while LDLR overexpression enhances Lp(a) clearance. Receptors such as VLDLR, LRP-1, and LRP-8 were not involved in this process [85]. These findings provide a mechanistic basis for PCSK9 inhibitors reducing Lp(a) through LDLR-mediated pathways and establish a foundation for targeted treatment of elevated Lp(a). Furthermore, genetic analysis by Cui et al. confirmed that specific variant genotypes of PCSK9 E670G (AG+GG) and LDLR rs688 (CT+TT) are significantly associated with reduced HDL-C levels and negatively correlated with hemodynamic parameters [86], further suggesting that genetic factors may influence the efficacy of PCSK9 inhibitors.

Regarding safety, PCSK9 inhibitors are generally well-tolerated. The most common adverse reactions are injection site reactions, occurring in approximately 10%-20% of cases, typically manifesting as local pain, erythema, pruritus, or induration. These are usually mild and self-limiting within days. Allergic reactions are relatively rare, with severe anaphylaxis being exceedingly uncommon; mild allergic symptoms generally resolve quickly after discontinuation. Long-term use has not demonstrated significant nephrotoxicity. Numerous clinical trials and observational studies indicate that while effectively lowering LDL-C, PCSK9 inhibitors do not adversely affect renal function, and no significant drug interactions have been observed when co-administered with immunosuppressants [87]. Although neurological adverse reactions and abnormal liver function have been reported, their incidence is low, and current evidence is insufficient to establish a direct causal relationship with PCSK9 inhibitors. Notably, beyond its role in lipid metabolism, PCSK9 may indirectly inhibit RNA virus replication and infection by binding to specific viral surface proteins, interfering with viral entry into host cells, or modulating host immune pathways [88]. This function suggests that PCSK9 may play a role in antiviral defense, and the use of PCSK9 inhibitors could potentially attenuate this protective effect, increasing the risk of specific viral infections, a concern requiring particular attention in immunocompromised renal transplant recipients.

### 4.2. Advantages of PCSK9 inhibitors compared with statins

In the management of dyslipidemia in renal transplant recipients, PCSK9 inhibitors offer three core advantages compared to conventional statins. First, PCSK9 inhibitors provide superior lipid-lowering efficacy without drug interaction limitations. Most statins (except pravastatin and fluvastatin) require specific metabolic pathways and may lead to blood concentration fluctuations when co-administered with immunosuppressants commonly used in transplant recipients. In contrast, PCSK9 inhibitors do not rely on these pathways, allowing safe combination with immunosuppressants and achieving significantly greater LDL-C reduction as monotherapy. Second, PCSK9 inhibitors demonstrate improved safety and tolerability. Statins frequently cause adverse effects such as myalgia, abnormal liver function, and increased diabetes risk, often necessitating dose reduction or discontinuation in transplant patients. Adverse reactions to PCSK9 inhibitors are primarily mild injection site reactions, with no conclusive evidence of hepatorenal toxicity or increased diabetes risk. Third, PCSK9 inhibitors have broader applicability. For transplant recipients who are statin-intolerant, fail to achieve lipid targets despite maximum tolerated statin plus ezetimibe therapy, or require stringent lipid control due to high ASCVD risk, PCSK9 inhibitors serve as an effective alternative or add-on therapy, addressing the limitations of conventional lipid-lowering strategies.

Furthermore, regarding comprehensive lipid modulation, PCSK9 inhibitors offer an advantage not seen with statins by modulating Lp(a) levels. Specific PCSK9 inhibitors can moderately reduce Lp(a), whereas statins typically cause a mild increase. This difference is particularly important for renal transplant recipients: this population often has elevated baseline Lp(a) levels and increased ASCVD risk, and the Lp(a)-lowering effect of PCSK9 inhibitors may further reduce their cardiovascular event risk. Mechanistically, the modulation of Lp(a) by PCSK9 inhibitors is linked to the LDLR-mediated clearance pathway, acting synergistically with their overall lipid-lowering efficacy. In contrast, the statin-induced increase in Lp(a) is potentially related to a compensatory response following cholesterol synthesis inhibition, for which there is currently no effective mitigation strategy. This characteristic further supports the adjunctive therapeutic value of PCSK9 inhibitors in renal transplant recipients, especially those with elevated Lp(a).

It must be emphasized that the advantages of PCSK9 inhibitors do not negate the value of statins. Statins remain the first-line foundational therapy for lipid management in renal transplant recipients. High-intensity statin therapy can achieve LDL-C reduction comparable to some PCSK9 inhibitors in the general population, and high-dose statins are still the preferred recommendation for transplant recipients with good tolerance and no significant drug interactions. More importantly, statins possess established anti-inflammatory effects, providing independent long-term cardiovascular protection and delaying the progression of atherosclerotic inflammation by inhibiting macrophage activation and reducing C-reactive protein levels; currently, there is insufficient evidence to indicate that PCSK9 inhibitors have direct anti-inflammatory effects. Therefore, these two drug classes are not substitutive but complementary. Clinical practice requires individualized lipid management strategies based on patient tolerance, achievement of lipid targets, and the risk of drug interactions.

### 4.3. Adverse reactions and safety

#### 4.3.1. Common adverse reactions and monitoring

The most common adverse reactions to PCSK9 inhibitors are injection site reactions. Although typically mild and self-limiting, they may impact patient medication adherence. During clinical use, physicians should inform patients of the possibility of such reactions in advance and provide nursing advice, such as appropriate cold compress before and after injection, and rotating injection sites to avoid repeated stimulation at the same location, thereby alleviating discomfort.

Although the incidence of allergic reactions is low, vigilance remains necessary. Clinical studies indicate that some patients may experience allergic symptoms such as rash, pruritus, or dyspnea following PCSK9 inhibitor use. Severe allergic reactions are exceedingly rare, and mild symptoms typically resolve rapidly after discontinuation. Therefore, physicians should thoroughly review patients’ allergy history when prescribing, enhance monitoring during initial treatment, and promptly identify and manage potential allergic reactions. Regarding neurological adverse reactions, their underlying mechanism is not fully elucidated and may be related to the role of PCSK9 in the nervous system. Although current evidence does not clearly indicate that PCSK9 inhibitors significantly increase the risk of such adverse reactions, regular neurological assessments and long-term follow-up are still recommended for patients with a history of neurological disorders to ensure medication safety. Reports of abnormal liver function are relatively infrequent but still warrant attention. Some patients may experience elevated liver enzymes following PCSK9 inhibitor use, potentially related to drug metabolism pathways [89]. In clinical practice, it is advisable to assess liver function prior to treatment and monitor liver function parameters regularly during therapy, enabling timely detection and management of abnormalities. This approach ensures medication safety and allows physicians to adjust treatment plans as needed to mitigate potential hepatotoxicity risks.

In summary, to ensure patient safety, clinicians should regularly monitor biochemical parameters, including liver function, renal function, and creatine kinase levels, to promptly identify potential adverse reactions. Although PCSK9 inhibitors demonstrate an overall favorable safety profile, comprehensive monitoring during treatment remains essential to safeguard patient health.

#### 4.3.2. Long-term use safety and virus infection risk

First, PCSK9 inhibitors demonstrate a favorable safety profile when co-administered with immunosuppressants. Studies indicate that PCSK9 inhibitors such as alirocumab and evolocumab do not significantly affect the blood concentrations of immunosuppressants. For example, a study in patients with anti-HMGCR-positive immune-mediated necrotizing myopathy showed that treatment with PCSK9 inhibitors did not lead to decreased muscle strength; instead, creatine kinase levels improved [90]. Metabolically, PCSK9 inhibitors are not processed by the liver or kidneys and do not share the CYP3A4 or CYP2C9 metabolic pathways with mainstream immunosuppressants, thus avoiding interference with immunosuppressant blood levels and potentially offering additional clinical benefits in some cases. Second, long-term use of PCSK9 inhibitors does not lead to significant renal function decline. Multiple clinical trials and observational studies confirmed that PCSK9 inhibitors effectively lower LDL-C without adversely affecting patient renal function. For example, a study involving 933 patients with coronary heart disease found that although the LDL-C target achievement rate was lower in patients with renal impairment (eGFR <60 mL/min/1.73 m^2^) compared to those with normal renal function, the addition of PCSK9 inhibitors significantly improved their LDL-C target attainment rate [91], further confirming the renal safety of PCSK9 inhibitors. Finally, PCSK9 inhibitors are associated with a low incidence of adverse reactions, with no severe adverse events observed. A recent meta-analysis indicated no significant differences in the incidence of treatment-related adverse events, serious adverse events, or diabetes-related adverse events between the PCSK9 inhibitor group and the control group [92]. Although early studies raised concerns about potential neurocognitive adverse effects, subsequent large-scale clinical trials have demonstrated that such events are extremely rare. In summary, PCSK9 inhibitors exhibit a favorable long-term safety profile and are suitable for lipid management in renal transplant patients at high cardiovascular risk.

Recent studies have found that beyond its role in lipid metabolism, PCSK9 may also indirectly inhibit RNA virus replication and infection by binding to specific viral surface proteins, interfering with viral entry into host cells, or modulating host immune pathways [93], suggesting a potential role in antiviral defense. The use of PCSK9 inhibitors might attenuate this protective effect, increasing the risk of specific viral infections. In the general population, small-scale clinical observations indicate that although the incidence of RNA viral infections, such as influenza and SARS-CoV-2, in patients receiving long-term PCSK9 inhibitor therapy (e.g., evolocumab, alirocumab) shows no significant difference compared to the placebo group, there is a trend toward prolonged symptom duration and a slight increase in the risk of severe disease [94]. In contrast, renal transplant recipients, due to long-term use of immunosuppressants such as calcineurin inhibitors (e.g., cyclosporine A, tacrolimus) and glucocorticoids, are inherently immunocompromised and exhibit significantly reduced antiviral capacity compared to the general population [95]. When combined with PCSK9 inhibitors, the immunocompromised state and the potential impairment of PCSK9-mediated antiviral functions may have a synergistic effect, further increasing the risk of infection [96]. Although dedicated studies on the risk of viral infection in transplant recipients receiving PCSK9 inhibitor therapy are currently limited, a case report by García-Agudo et al. described a 74-year-old male renal transplant recipient who switched to alirocumab due to atorvastatin allergy. During treatment, his lipid levels were well-controlled, everolimus blood concentration remained stable, tolerance was good, and no viral infection occurred [97]. While this case does not establish a direct causal link between PCSK9 inhibitor use and viral infection, it provides a reference for clinical vigilance.

Therefore, in clinical practice, stricter viral monitoring measures are required for renal transplant recipients receiving PCSK9 inhibitors: pretreatment screening for antibodies and viral loads of common viruses such as CMV and EBV; repeat viral load testing every 3–6 months during treatment; and enhanced prevention against respiratory viruses like influenza and SARS-CoV-2 (e.g., vaccination). If patients present with suspected viral infection symptoms such as fever, fatigue, or pulmonary infection, temporary discontinuation of the PCSK9 inhibitor is advised, along with prompt initiation of antiviral therapy to prevent disease progression and compromise graft outcomes. Future multi-center, large-scale prospective cohort studies are needed to quantify the association between PCSK9 inhibitor use and viral infection risk in this population, while also exploring individualized strategies (e.g., intermittent dosing regimens based on viral load) to optimize lipid management while ensuring medication safety.

### 4.4. Challenges and future directions

#### 4.4.1. Effect of Lp (a) on the efficacy of PCSK9 inhibitors and its countermeasures

For renal transplant recipients considered for PCSK9 inhibitor therapy, baseline Lp(a) testing and LDLR gene screening are recommended prior to treatment. For patients with elevated Lp(a) (particularly ≥125 nmol/L) or those carrying LDLR mutations, the potential for reduced efficacy of PCSK9 inhibitors should be anticipated, and an individualized plan should be developed. This may include combining with other lipid-lowering agents, adjusting the PCSK9 inhibitor dosage, or selecting Lp(a)-targeted therapies. If the lipid-lowering response falls short of expectations after treatment initiation, genetic testing and Lp(a) measurement should be promptly conducted to clarify the cause of suboptimal efficacy and identify alternative therapeutic targets, thereby avoiding delays in achieving lipid management goals [98].

Furthermore, the differential effects of PCSK9 inhibitors and statins on Lp(a) require particular attention: PCSK9 inhibitors moderately reduce Lp(a) *via* the LDLR-mediated pathway, whereas statins (especially at moderate to high doses) typically cause a mild increase in Lp(a). For renal transplant recipients requiring low-dose statin therapy (e.g., those with statin intolerance but still needing foundational treatment), the statin-induced elevation in Lp(a) may compound with Lp(a)’s inherent competition for LDLR binding sites, further attenuating the LDL-C-lowering efficacy of PCSK9 inhibitors. Therefore, before initiating PCSK9 inhibitor therapy, in addition to assessing baseline Lp(a) and LDLR genotype, it is essential to clarify the patient’s current statin use. If the patient is already on statins and exhibits a rising trend in Lp(a), switching to a regimen combining a PCSK9 inhibitor with ezetimibe is recommended. This strategy avoids the adverse effect of statins on Lp(a), while ezetimibe’s inhibition of intestinal cholesterol absorption synergizes with the PCSK9 inhibitor, ensuring the achievement of lipid control targets [99].

#### 4.4.2. Other challenges and future exploration direction

##### 4.4.2.1. Core challenges

The first is economic constraints. Compared to statins (e.g., atorvastatin) and ezetimibe, PCSK9 inhibitors entail significantly higher treatment costs, limiting their widespread clinical use. Substantial variations in healthcare policies and drug pricing across different countries and regions further exacerbate disparities in the clinical accessibility of PCSK9 inhibitors. In some areas, PCSK9 inhibitors are only covered by health insurance for high-risk ASCVD patients, excluding renal transplant recipients. Consequently, this population must bear the full cost of treatment out-of-pocket, leading to a substantial increase in financial burden and preventing many eligible patients from accessing this effective lipid-lowering therapy.

Although the treatment cost of PCSK9 inhibitors is substantially higher than that of conventional statins, which somewhat limits their broad application, their economic evaluation should be considered within the overall treatment landscape for refractory hyperlipidemia. For kidney transplant recipients who are statin-intolerant or have refractory disease, other advanced treatment options, such as therapeutic apheresis (including specific LDL apheresis or Lp(a) apheresis), are also considerably expensive. Apheresis requires patients to undergo this invasive procedure regularly (e.g., weekly or bi-weekly) in a clinical setting, with each session lasting several hours. The annual total cost can be comparable to that of PCSK9 inhibitors, yet apheresis imposes a significantly greater burden on patients and carries additional procedure-related risks, such as vascular access complications [100]. Indeed, studies have demonstrated that PCSK9 inhibitors can effectively reduce or even eliminate the need for apheresis in some patients, suggesting potential long-term cost-saving benefits [101]. Pharmacoeconomic studies indicate that the cost-effectiveness of PCSK9 inhibitors in high-risk populations is closely tied to their price, and their value in preventing cardiovascular events becomes fully realized following price optimization [102]. Furthermore, novel lipid-lowering agents such as inclisiran, despite requiring only biannual injections, are also positioned within the high-cost tier of therapies [103,104]. Therefore, from a long-term pharmacoeconomic perspective, PCSK9 inhibitors represent a valuable alternative therapeutic option, owing to their convenient administration, potent efficacy in lipid reduction, and proven cardiovascular benefits. Future drug pricing strategies and healthcare reimbursement policies should more fully reflect their holistic value in improving patient outcomes and quality of life.

The second is limited clinical evidence. Current research on the use of PCSK9 inhibitors in renal transplant recipients is predominantly based on small-sample cohort studies and case reports, lacking support from high-quality evidence derived from large-scale, multicenter randomized controlled trials (RCTs). Published studies generally suffer from small-sample sizes and short follow-up durations, preventing a comprehensive and reliable assessment of the long-term cardiovascular benefits of PCSK9 inhibitors and their potential impact on graft function in this population. Furthermore, safety and efficacy data for PCSK9 inhibitors in specific subgroups, such as recipients with hepatitis B/C coinfection or elderly transplant patients, are extremely scarce, significantly hindering the development of individualized and precise treatment strategies for these subpopulations.

##### 4.4.2.2. Future research directions

First, we can conduct dedicated clinical trials for renal transplant recipients. Multicenter, large-sample RCTs should be designed to clarify the optimal dosage, dosing frequency, and long-term impact on graft function and cardiovascular outcomes of different types of PCSK9 inhibitors (monoclonal antibodies, small interfering RNA, ultra-long-acting monoclonal antibodies) in renal transplant recipients, providing high-level evidence for clinical guidelines.

Second, we can optimize economic solutions, encouraging pharmaceutical innovation to reduce production costs while promoting healthcare policy reforms to include PCSK9 inhibitors for renal transplant recipients in medical insurance reimbursement schemes. Concurrently, we can utilize centralized procurement and bulk tendering mechanisms to lower drug prices and enhance clinical accessibility.

Third, we can expand research on special subgroups, conducting studies on the safety and efficacy of PCSK9 inhibitors in special subgroups, such as renal transplant recipients with comorbidities (e.g., diabetes, hepatitis B or C), elderly recipients, and pediatric recipients, to identify contraindications and dose adjustment strategies for specific subpopulations and avoid a one-size-fits-all treatment approach.

## 5. Conclusion

PCSK9 inhibitors demonstrate favorable efficacy and safety in lipid management for kidney transplant recipients, offering a valuable supplementary option for patients with statin intolerance, suboptimal response, or significant drug interactions. However, it must be emphasized that statins remain the first-line foundational therapy. High-intensity statin regimens can achieve lipid-lowering efficacy comparable to some PCSK9 inhibitors in appropriate populations, and their well-established anti-inflammatory effects provide irreplaceable benefits for cardiovascular event prevention. In future clinical practice, statins (including combination therapy with ezetimibe) should be prioritized, with PCSK9 inhibitors reserved for cases where statins alone are insufficient, thereby establishing a stepped treatment approach of foundational therapy supplemented by advanced options. Current evidence supporting PCSK9 inhibitors is largely based on small-sample case reports or short-term cohort studies, lacking validation from large-scale multicenter randomized controlled trials regarding long-term cardiovascular benefits and impacts on allograft outcomes. Dedicated studies are needed to address these evidence gaps, while further exploration of the efficacy and safety of combining statins with PCSK9 inhibitors will enable more precise and personalized lipid management strategies for kidney transplant recipients.

## Data Availability

Data sharing is not applicable to this article as no datasets were generated or analyzed during the current study.

## References

[1] Stoumpos S, Jardine AG, Mark PB. Cardiovascular morbidity and mortality after kidney transplantation. Transpl Int. 2015;28(1):10–21. doi: 10.1111/tri.12413. 25081992

[2] United States Renal Data System. 2024. USRDS annual data report: epidemiology of kidney disease in the United States. Bethesda, MD: National Institutes of Health, National Institute of Diabetes and Digestive and Kidney Diseases, 2024.

[3] Wu H, Ballantyne CM. Dyslipidaemia: PCSK9 inhibitors and foamy monocytes in familial hypercholesterolaemia. Nat Rev Cardiol. 2017;14(7):385–386. doi: 10.1038/nrcardio.2017.75. 28492287 PMC5808934

[4] Feinstein MJ, Hsue PY, Benjamin LA, et al. Characteristics, prevention, and management of cardiovascular disease in people living with HIV: a scientific statement from the American Heart Association. Circulation. 2019;140(2):e98–e124. doi: 10.1161/CIR.0000000000000695. 31154814 PMC7993364

[5] Caughey GE, Gabb GM, Ronson S, et al. Association of statin exposure with histologically confirmed idiopathic inflammatory myositis in an Australian population. JAMA Intern Med. 2018;178(9):1224–1229. doi: 10.1001/jamainternmed.2018.2859. 30073275 PMC6142971

[6] Bock ME, Wall L, Dobrec C, et al. Management of dyslipidemia in pediatric renal transplant recipients. Pediatr Nephrol. 2021;36(1):51–63. doi: 10.1007/s00467-019-04428-y. 31897714

[7] Org E, Blum Y, Kasela S, et al. Relationships between gut microbiota, plasma metabolites, and metabolic syndrome traits in the METSIM cohort. Genome Biol. 2017;18(1):70. doi: 10.1186/s13059-017-1194-2. 28407784 PMC5390365

[8] Schloss MJ, Swirski FK, Nahrendorf M. Modifiable cardiovascular risk, hematopoiesis, and innate immunity. Circ Res. 2020;126(9):1242–1259. doi: 10.1161/CIRCRESAHA.120.315936. 32324501 PMC7185037

[9] Ferrannini E, Solini A, Baldi S, et al. Role of glycosuria in SGLT2 inhibitor-induced cardiorenal protection: a mechanistic analysis of the CREDENCE trial. Diabetes. 2024;73(2):250–259. doi: 10.2337/db23-0448. 37939214 PMC10796302

[10] Borges BC, Han X, Allen SJ, et al. Insulin signaling in LepR cells modulates fat and glucose homeostasis independent of leptin. Am J Physiol Endocrinol Metab. 2019;316(1):E121–E134. doi: 10.1152/ajpendo.00287.2018. 30376348 PMC6417687

[11] Luan X, Tian X, Zhang H, et al. Exercise as a prescription for patients with various diseases. J Sport Health Sci. 2019;8(5):422–441. doi: 10.1016/j.jshs.2019.04.002. 31534817 PMC6742679

[12] Taylor F, Combes G, Hare J. Improving clinical skills to support the emotional and psychological well-being of patients with end-stage renal disease: a qualitative evaluation of two interventions. Clin Kidney J. 2016;9(3):516–524. doi: 10.1093/ckj/sfw017. 27274842 PMC4886913

[13] Perumpail BJ, Khan MA, Yoo ER, et al. Clinical epidemiology and disease burden of nonalcoholic fatty liver disease. World J Gastroenterol. 2017;23(47):8263–8276. doi: 10.3748/wjg.v23.i47.8263. 29307986 PMC5743497

[14] Kuzmich N, Andresyuk E, Porozov Y, et al. PCSK9 as a target for development of a new generation of hypolipidemic drugs. Molecules. 2022;27(2):434. doi: 10.3390/molecules27020434. 35056760 PMC8778893

[15] Zhu D, Qin H, Wang X, et al. Discovery of [5,5′-bibenzo[d][1,3]dioxol]-6-substituted amine derivatives as potent proprotein convertase subtilisin/kexin type 9 inhibitors. Chem Biol Drug Des. 2023;102(1):153–167. doi: 10.1111/cbdd.14264. 37170061

[16] Castellano-Castillo D, Núñez-Sánchez MÁ, Balaguer-Román A, et al. The role of PCSK9 in metabolic dysfunction-associated steatotic liver disease and its impact on bariatric surgery outcomes. Surg Obes Relat Dis. 2024;20(7):652–659. doi: 10.1016/j.soard.2024.01.017. 38490825

[17] Los B, Ferreira GM, Borges JB, et al. Effects of PCSK9 missense variants on molecular conformation and biological activity in transfected HEK293FT cells. Gene. 2023;851:146979. doi: 10.1016/j.gene.2022.146979. 36261084

[18] Alleyne C, Amin RP, Bhatt B, et al. Series of novel and highly potent cyclic peptide PCSK9 inhibitors derived from an mRNA display screen and optimized via structure-based design. J Med Chem. 2020;63(22):13796–13824. doi: 10.1021/acs.jmedchem.0c01084. 33170686

[19] Sgrignani J, Fassi EMA, Lammi C, et al. Exploring proprotein convertase subtilisin/kexin 9 (PCSK9) autoproteolysis process by molecular simulations: hints for drug design. ChemMedChem. 2020;15(16):1601–1607. doi: 10.1002/cmdc.202000431. 32558225

[20] Haas ME, Levenson AE, Sun X, et al. The role of proprotein convertase subtilisin/kexin type 9 in nephrotic syndrome-associated hypercholesterolemia. Circulation. 2016;134(1):61–72. doi: 10.1161/CIRCULATIONAHA.115.020912. 27358438 PMC5345853

[21] Bourbiaux K, Legrand B, Verdié P, et al. Potent lys patch-containing stapled peptides targeting PCSK9. J Med Chem. 2021;64(15):10834–10848. doi: 10.1021/acs.jmedchem.0c02051. 34266235

[22] Charbe NB, Zacconi FC, Kowthavarapu VK, et al. Targeting allosteric site of PCSK9 enzyme for the identification of small molecule inhibitors: an in silico drug repurposing study. Biomedicines. 2024;12(2):286. doi: 10.3390/biomedicines12020286. 38397888 PMC10887305

[23] Chen Z, Shao W, Li Y, et al. Inhibition of PCSK9 prevents and alleviates cholesterol gallstones through PPARα-mediated CYP7A1 activation. Metabolism. 2024;152:155774. doi: 10.1016/j.metabol.2023.155774. 38191052

[24] Rannikko J, Jacome Sanz D, Ortutay Z, et al. Reduced plasma PCSK9 response in patients with bacteraemia is associated with mortality. J Intern Med. 2019;286(5):553–561. doi: 10.1111/joim.12946. 31166632

[25] Marfella R, Prattichizzo F, Sardu C, et al. Evidence of an anti-inflammatory effect of PCSK9 inhibitors within the human atherosclerotic plaque. Atherosclerosis. 2023;378:117180. doi: 10.1016/j.atherosclerosis.2023.06.971. 37422356

[26] Zhan QY, Xie LX, Wang C. [Promoting critical care system and capacity building in pulmonary and critical care medicine subspecialties]. Zhonghua Yi Xue Za Zhi. 2023;103(40):3149–3151. doi: 10.3760/cma.j.cn112137-20230602-00919. 37879866

[27] Katzmann JL, Laufs U. PCSK9-directed therapies: an update. Curr Opin Lipidol. 2024;35(3):117–125. doi: 10.1097/MOL.0000000000000919. 38277255

[28] Liu C, Chen J, Chen H, et al. PCSK9 inhibition: from current advances to evolving future. Cells. 2022;11(19):2972. doi: 10.3390/cells11192972. 36230934 PMC9562883

[29] Grześk G, Dorota B, Wołowiec Ł, et al. Safety of PCSK9 inhibitors. Biomed Pharmacother. 2022;156:113957. doi: 10.1016/j.biopha.2022.113957. 36411665

[30] Dong Z, Zhang W, Chen S, et al. Silibinin A decreases statin‑induced PCSK9 expression in human hepatoblastoma HepG2 cells. Mol Med Rep. 2019;20(2):1383–1392. doi: 10.3892/mmr.2019.10344. 31173243

[31] Paryani M, Gupta N, Jain SK, et al. Lowering LDL cholesterol by PCSK9 inhibition: a new era of gene silencing, RNA, and alternative therapies. Naunyn Schmiedebergs Arch Pharmacol. 2025;398(6):6597–6615. doi: 10.1007/s00210-025-03826-4. 39883121

[32] Gouverneur A, Sanchez-Pena P, Veyrac G, et al. Neurocognitive disorders associated with PCSK9 inhibitors: a pharmacovigilance disproportionality analysis. Cardiovasc Drugs Ther. 2023;37(2):271–276. doi: 10.1007/s10557-021-07242-7. 34436707

[33] Xiang Y, Gan L, Du H, et al. Cost-effectiveness of adding ezetimibe and/or PCSK9 inhibitors to high-dose statins for secondary prevention of cardiovascular disease in Chinese adults. Int J Technol Assess Health Care. 2023;39(1):e53. doi: 10.1017/S0266462323000296. 37650314 PMC11570136

[34] Liou JW, Chen PY, Gao WY, et al. Natural phytochemicals as small-molecule proprotein convertase subtilisin/kexin type 9 inhibitors. Tzu Chi Med J. 2024;36(4):360–369. doi: 10.4103/tcmj.tcmj_46_24. 39421488 PMC11483095

[35] Katzmann JL, Custodis F, Schirmer SH, et al. [Update on PCSK9 inhibition]. Herz. 2022;47(3):196–203. doi: 10.1007/s00059-022-05112-y. 35445838

[36] German CA, Shapiro MD. Small interfering RNA therapeutic inclisiran: a new approach to targeting PCSK9. BioDrugs. 2020;34(1):1–9. doi: 10.1007/s40259-019-00399-6. 31782112

[37] Manzato M, Wright RS, Jaffe AS, et al. Lipoprotein (a): underrecognized Risk with a Promising Future. Rev Cardiovasc Med. 2024;25(11):393. doi: 10.31083/j.rcm2511393. 39618878 PMC11607505

[38] Wright RS, Ray KK, Landmesser U, et al. Effects of inclisiran in patients with atherosclerotic cardiovascular disease: a pooled analysis of the ORION-10 and ORION-11 randomized trials. Mayo Clin Proc. 2024;99(8):1222–1235. doi: 10.1016/j.mayocp.2024.03.025. 39093262

[39] Cheng Z, Gao M, Liu Y, et al. Safety and efficacy of inclisiran in treating hypercholesterolemia: a systemic review and meta-analysis. Nutr Metab Cardiovasc Dis. 2025;35(5):103779. doi: 10.1016/j.numecd.2024.10.017. 39674726

[40] Marrs JC, Anderson SL. Inclisiran for the treatment of hypercholesterolaemia. Drugs Context. 2024;13:1–9. doi: 10.7573/dic.2023-12-3. PMC11619601 39640378

[41] Mikami T, Fujiwara Y, Akahori M[, et al. Pharmacological and clinical profile of inclisiran sodium, a long-acting LDL cholesterol lowering siRNA, LEQVIO for s.c. injection syringe 300 mg. Nihon Yakurigaku Zasshi. 2024;159(4):254–263. doi: 10.1254/fpj.24018. 38945909

[42] Xu M, Zhu X, Wu J, et al. PCSK9 inhibitor recaticimab for hypercholesterolemia on stable statin dose: a randomized, double-blind, placebo-controlled phase 1b/2 study. BMC Med. 2022;20(1):13. doi: 10.1186/s12916-021-02208-w. 35039035 PMC8763618

[43] Xu M, Wang Z, Zhang Y, et al. Recaticimab monotherapy for nonfamilial hypercholesterolemia and mixed hyperlipemia: the phase 3 REMAIN-1 randomized trial. J Am Coll Cardiol. 2024;84(20):2026–2036. doi: 10.1016/j.jacc.2024.07.035. 39387764

[44] Hermel M, Chiou A, Minhas AMK, et al. Highlights of cardiovascular disease prevention studies presented at the 2023 American Heart Association scientific sessions. Curr Atheroscler Rep. 2024;26(4):119–131. doi: 10.1007/s11883-024-01193-8. 38441801

[45] Sun Y, Lv Q, Guo Y, et al. Recaticimab as add-on therapy to statins for nonfamilial hypercholesterolemia: the randomized, phase 3 REMAIN-2 trial. J Am Coll Cardiol. 2024;84(20):2037–2047. doi: 10.1016/j.jacc.2024.09.012. 39505412

[46] Gupta K, Hinkamp C, Andrews T, et al. Highlights of cardiovascular disease prevention studies presented at the 2023 European Society of Cardiology Congress. Curr Atheroscler Rep. 2023;25(12):965–978. doi: 10.1007/s11883-023-01164-5. 37975955

[47] Şener YZ, Tokgözoğlu L. Pleiotropy of PCSK9: functions in extrahepatic tissues. Curr Cardiol Rep. 2023;25(9):979–985. doi: 10.1007/s11886-023-01918-2. 37428313

[48] Ballantyne CM, Banka P, Mendez G, et al. Phase 2b randomized trial of the oral PCSK9 inhibitor MK-0616. J Am Coll Cardiol. 2023;81(16):1553–1564. doi: 10.1016/j.jacc.2023.02.018. 36889610

[49] Faluk M, Wardhere A, Mohamoud A, et al. Evolution of LDL-C lowering medications and their cardiovascular benefits: past, present, and future. Curr Probl Cardiol. 2024;49(7):102637. doi: 10.1016/j.cpcardiol.2024.102637. 38735347

[50] Raal F, Fourie N, Scott R, et al. Long-term efficacy and safety of lerodalcibep in heterozygous familial hypercholesterolaemia: the LIBerate-HeFH trial. Eur Heart J. 2023;44(40):4272–4280. doi: 10.1093/eurheartj/ehad596. 37639462 PMC10590131

[51] Giugliano RP, Pedersen TR, Saver JL, et al. Stroke prevention with the PCSK9 (proprotein convertase subtilisin-kexin type 9) inhibitor evolocumab added to statin in high-risk patients with stable atherosclerosis. Stroke. 2020;51(5):1546–1554. doi: 10.1161/STROKEAHA.119.027759. 32312223

[52] Jin P, Gao D, Cong G, et al. Role of PCSK9 in homocysteine-accelerated lipid accumulation in macrophages and atherosclerosis in ApoE-/- mice. Front Cardiovasc Med. 2021;8:746989. PMID: 34660746; PMCID: PMC8517151. doi: 10.3389/fcvm.2021.746989. 34660746 PMC8517151

[53] Wang W, Luo R, Pei D, et al. Association of serum PCSK9 levels with platelet function in patients with acute coronary syndromes. Medicine (Baltimore). 2023;102(15):e33026. PMID: 37058054; PMCID: PMC10101279. doi: 10.1097/MD.0000000000033026. 37058054 PMC10101279

[54] Song L, Zhao X, Chen R, et al. Association of PCSK9 with inflammation and platelet activation markers and recurrent cardiovascular risks in STEMI patients undergoing primary PCI with or without diabetes. Cardiovasc Diabetol. 2022;21(1):80. PMID: 35596184; PMCID: PMC9123773. doi: 10.1186/s12933-022-01519-3. 35596184 PMC9123773

[55] Wang S, Fu D, Liu H, et al. Independent association of PCSK9 with platelet reactivity in subjects without statin or antiplatelet agents. Front Cardiovasc Med. 2022;9:934914. PMID: 36324757; PMCID: PMC9618652. doi: 10.3389/fcvm.2022.934914. 36324757 PMC9618652

[56] Ziogos E, Chelko SP, Harb T, et al. Platelet activation and endothelial dysfunction biomarkers in acute coronary syndrome: the impact of PCSK9 inhibition. Eur Heart J Cardiovasc Pharmacother. 2023;9(7):636–646. PMID: 37468450; PMCID: PMC12098939. doi: 10.1093/ehjcvp/pvad051. 37468450 PMC12098939

[57] Laudette M, Lindbom M, Arif M, et al. Cardiomyocyte-specific PCSK9 deficiency compromises mitochondrial bioenergetics and heart function. Cardiovasc Res. 2023;119(7):1537–1552. PMID: 36880401; PMCID: PMC10318396. doi: 10.1093/cvr/cvad041. 36880401 PMC10318396

[58] Zhang X, Xu H, Yu J, et al. Immune regulation of the liver through the PCSK9/CD36 pathway during heart transplant rejection. Circulation. 2023;148(4):336–353. PMID: 37232170. doi: 10.1161/CIRCULATIONAHA.123.062788. 37232170

[59] Doiron S, Paquette M, Baass A, et al. Association between circulating PCSK9 and proteinuria in nephrotic syndrome: a cross-sectional study. Clin Biochem. 2022;109-110:51–56. PMID: 35940295. doi: 10.1016/j.clinbiochem.2022.08.002. 35940295

[60] Hummelgaard S, Kresse JC, Jensen MS, et al. Emerging roles of PCSK9 in kidney disease: lipid metabolism, megalin regulation and proteinuria. Pflugers Arch. 2025;477(6):773–786. PMID: 39964484. doi: 10.1007/s00424-025-03069-5. 39964484

[61] Ahamad S, Mathew S, Khan WA, et al. Development of small-molecule PCSK9 inhibitors for the treatment of hypercholesterolemia. Drug Discov Today. 2022;27(5):1332–1349. doi: 10.1016/j.drudis.2022.01.014. 35121175

[62] Xiang Q, Liu WF, Zeng JL, et al. Effect of PCSK9 on vascular smooth muscle cell functions: a new player in atherosclerosis. Curr Med Chem. 2021;28(36):7446–7460. doi: 10.2174/0929867328666210531150302. 34060998

[63] Jeswani BM, Sharma S, Rathore SS, et al. PCSK9 inhibitors: the evolving future. Health Sci Rep. 2024;7(11):e70174. doi: 10.1002/hsr2.70174. 39479289 PMC11522611

[64] Nishikido T, Ray KK. Targeting the peptidase PCSK9 to reduce cardiovascular risk: implications for basic science and upcoming challenges. Br J Pharmacol. 2021;178(11):2168–2185. doi: 10.1111/bph.14851. 31465540

[65] Chen T, Wang Z, Xie J, et al. Efficacy and safety of PCSK9 inhibitors in patients with diabetes: a systematic review and meta-analysis. Nutr Metab Cardiovasc Dis. 2023;33(9):1647–1661. doi: 10.1016/j.numecd.2023.05.033. 37414664

[66] Imbalzano E, Ilardi F, Orlando L, et al. The efficacy of PCSK9 inhibitors on major cardiovascular events and lipid profile in patients with diabetes: a systematic review and meta-analysis of randomized controlled trials. Eur Heart J Cardiovasc Pharmacother. 2023;9(4):318–327. doi: 10.1093/ehjcvp/pvad019. 36972610

[67] Moshkani Farahani M, Nasiri A, Salari M, et al. The therapeutic effect of PCSK9 inhibitors on dyslipidemia: one-year follow up. Eur J Transl Myol. 2024;34(3):12937. https://pubmed.ncbi.nlm.nih.gov/39283139/ doi: 10.4081/ejtm.2024.12937. 39283139 PMC11487668

[68] García-Agudo R, Rojas-Fernández MÁ, Canllavi-Fiel E, et al. Safe and successful treatment with Pcsk9 inhibitors in hypercholesterolemia and renal transplantation: a case report. Transplant Proc. 2023;55(8):1921–1923. doi: 10.1016/j.transproceed.2023.07.020. 37612152

[69] Choi HD, Kim JH. An updated meta-analysis for safety evaluation of alirocumab and evolocumab as PCSK9 inhibitors. Cardiovasc Ther. 2023;2023:7362551. doi: 10.1155/2023/7362551. 36704607 PMC9834631

[70] Ordóñez-Fernández L, Rodríguez-Ferreras A, Carriles C, et al. Pneumonia in a patient with kidney transplant treated with alirocumab and everolimus. Neumonía en un paciente con trasplante renal tratado con alirocumab y everolimus. Farm Hosp. 2019;43(2):74–76. doi: 10.7399/fh.11138. 30848181

[71] Lv P, Li Y, Wu L, et al. PCSK9 inhibitors in a renal transplant patient complicated with hepatitis B: a case report and literature review. Front Cardiovasc Med. 2022;9:937474. doi: 10.3389/fcvm.2022.937474. 36419496 PMC9676271

[72] Papasotiriou M, Ntrinias T, Savvidaki E, et al. Treatment of mixed dyslipidemia with alirocumab in a kidney transplant recipient: a case report. Transplant Proc. 2021;53(9):2775–2778. doi: 10.1016/j.transproceed.2021.08.027. 34602294

[73] Warden BA, Kaufman T, Minnier J, et al. Use of PCSK9 inhibitors in solid organ transplantation recipients. JACC Case Rep. 2020;2(3):396–399. doi: 10.1016/j.jaccas.2019.09.026. 34317250 PMC8311616

[74] Amaro JM, Villanego F, Orellana CD, et al. Management of dyslipidemia with evolocumab in kidney transplant recipients. Transplantation. 2024;108(5):e74–e76. doi: 10.1097/TP.0000000000004942. 38317278

[75] Alotaibi T, Nagib AM, Denewar A, et al. Inhibition of proprotein convertase subtilisin/kexin-9 after kidney transplant: single-center experience among patients with high cardiovascular risk. Exp Clin Transplant. 2024;22(Suppl 1):315–322. doi: 10.6002/ect.MESOT2023.P111. 38385418

[76] Khan SU, Yedlapati SH, Lone AN, et al. PCSK9 inhibitors and ezetimibe with or without statin therapy for cardiovascular risk reduction: a systematic review and network meta-analysis. BMJ. 2022;377:e069116. doi: 10.1136/bmj-2021-069116. 35508321

[77] Raal FJ, Chilton R, Ranjith N, et al. PCSK9 inhibitors: from nature’s lessons to clinical utility. Endocr Metab Immune Disord Drug Targets. 2020;20(6):840–854. doi: 10.2174/1871530320666200213114138. 32053090

[78] Sabouret P, Farnier M, Puymirat E. [PCSK9 inhibitors: what place in the management of dyslipidemia?]. Presse Med. 2019;48(3 Pt 1):227–237. doi: 10.1016/j.lpm.2019.01.009. 30853281

[79] Kosmas CE, Skavdis A, Sourlas A, et al. Safety and tolerability of PCSK9 inhibitors: current insights. Clin Pharmacol. 2020;12:191–202. doi: 10.2147/CPAA.S288831. 33335431 PMC7737942

[80] Elis A, Daud W, Cohen G, et al. Treatment with anti-PCSK9 monoclonal Ab: experience from a lipid clinic in Israel. Isr Med Assoc J. 2022;24(11):763–767. 36436046

[81] Kam N, Perera K, Zomer E, et al. Inclisiran as adjunct lipid-lowering therapy for patients with cardiovascular disease: a cost-effectiveness analysis. Pharmacoeconomics. 2020;38(9):1007–1020. doi: 10.1007/s40273-020-00948-w. 32789593

[82] Mercep I, Strikic D, Hrabac P, et al. PCSK9 inhibition: from effectiveness to cost-effectiveness. Front Cardiovasc Med. 2024;11:1339487. doi: 10.3389/fcvm.2024.1339487. 38988669 PMC11234837

[83] Marco-Benedí V, Cenarro A, Laclaustra M, et al. Lipoprotein(a) in hereditary hypercholesterolemia: influence of the genetic cause, defective gene and type of mutation. Atherosclerosis. 2022;349:211–218. PMID: 34456049. doi: 10.1016/j.atherosclerosis.2021.08.009. 34456049

[84] Ferdinand KC, Nasser SA. PCSK9 inhibition: discovery, current evidence, and potential effects on LDL-C and Lp(a). Cardiovasc Drugs Ther. 2015;29(3):295–308. PMID: 26068408. doi: 10.1007/s10557-015-6588-3. 26068408

[85] Romagnuolo R, Scipione CA, Marcovina SM, et al. Roles of the low density lipoprotein receptor and related receptors in inhibition of lipoprotein(a) internalization by proprotein convertase subtilisin/kexin type 9. PLoS One. 2017;12(7):e0180869. PMID: 28750079; PMCID: PMC5531514. doi: 10.1371/journal.pone.0180869. 28750079 PMC5531514

[86] Cui J, Qiu Y, Kang N, et al. Correlations of PCSK9 and LDLR gene polymorphisms and serum PCSK9 levels with atherosclerosis and lipid metabolism in patients on maintenance hemodialysis. J Clin Pharmacol. 2023;63(12):1430–1437. PMID: 37563753. doi: 10.1002/jcph.2332. 37563753

[87] Fang H, Shi M, Wang C, et al. PCSK9 potentiates innate immune response to RNA viruses by preventing AIP4-mediated polyubiquitination and degradation of VISA/MAVS. Proc Natl Acad Sci U S A. 2025;122(8):e2412206122. PMID: 40233407; PMCID: PMC11874596. doi: 10.1073/pnas.2412206122. 40233407 PMC11874596

[88] Zhang Y, Gao F, Li L, et al. Porcine reproductive and respiratory syndrome virus antagonizes PCSK9’s antiviral effect via Nsp11 endoribonuclease activity. Viruses. 2020;12(6):655. PMID: 32560445; PMCID: PMC7354446. doi: 10.3390/v12060655. 32560445 PMC7354446

[89] Liu H. Association between PCSK9 inhibitors and acute kidney injury: a pharmacovigilance study. Front Pharmacol. 2024;15:1353848. doi: 10.3389/fphar.2024.1353848. 39148544 PMC11324468

[90] Tiniakou E, Rivera E, Mammen AL, et al. Use of proprotein convertase subtilisin/kexin type 9 inhibitors in statin-associated immune-mediated necrotizing myopathy: a case series. Arthritis Rheumatol. 2019;71(10):1723–1726. doi: 10.1002/art.40919. 31058470

[91] Zhang S, Li ZF, Shi HW, et al. Comparison of low-density lipoprotein cholesterol (LDL-C) goal achievement and lipid-lowering therapy in the patients with coronary artery disease with different renal functions. Front Cardiovasc Med. 2022;9:859567. doi: 10.3389/fcvm.2022.859567. 35620524 PMC9127229

[92] Coppinger C, Movahed MR, Azemawah V, et al. A comprehensive review of PCSK9 inhibitors. J Cardiovasc Pharmacol Ther. 2022;27:10742484221100107. doi: 10.1177/10742484221100107. 35593194

[93] Dioh W, Chabane M, Tourette C, et al. Testing the efficacy and safety of BIO101, for the prevention of respiratory deterioration, in patients with COVID-19 pneumonia (COVA study): a structured summary of a study protocol for a randomised controlled trial. Trials. 2021;22(1):42. PMID: 33430924; PMCID: PMC7797700. doi: 10.1186/s13063-020-04998-5. 33430924 PMC7797700

[94] Navarese EP, Podhajski P, Gurbel PA, et al. PCSK9 inhibition during the inflammatory stage of SARS-CoV-2 infection. J Am Coll Cardiol. 2023;81(3):224–234. PMID: 36653090; PMCID: PMC9842071. doi: 10.1016/j.jacc.2022.10.030. 36653090 PMC9842071

[95] Eisenga MF, Zelle DM, Sloan JH, et al. High serum PCSK9 is associated with increased risk of new-onset diabetes after transplantation in renal transplant recipients. Diabetes Care. 2017;40(7):894–901. PMID: 28461454. doi: 10.2337/dc16-2258. 28461454

[96] Melexopoulou C, Marinaki S, Oikonomou E, et al. PCSK9 and inflammatory biomarkers in the early post kidney transplantation period. Eur Rev Med Pharmacol Sci. 2021;25(14):4762–4772. PMID: 34337724. doi: 10.26355/eurrev_202107_26388. 34337724

[97] Luo B, Zhong S, Wang X, et al. Management of blood lipids in post-kidney transplant patients: a systematic review and network meta-analysis. Front Pharmacol. 2024;15:1440875. PMID: 39439889; PMCID: PMC11493609. doi: 10.3389/fphar.2024.1440875. 39439889 PMC11493609

[98] Gulpen AJW, Claessen RJM, Vanmolkot FHM. [PCSK9 inhibitors; who benefits from these new cholesterol-lowering drugs?] Ned Tijdschr Geneeskd. 2020;164:D4325. https://pubmed.ncbi.nlm.nih.gov/32186821/. 32186821

[99] Eloso J, Awad A, Zhao X, et al. PCSK9 inhibitor use and outcomes using concomitant lipid-lowering therapies in the Veterans Health Administration. Am J Med Open. 2023;9:100035. doi: 10.1016/j.ajmo.2023.100035. 39035055 PMC11256282

[100] Tokgozoglu L, Kayikcioglu M. Familial hypercholesterolemia: global burden and approaches. Curr Cardiol Rep. 2021;23(10):151. doi: 10.1007/s11886-021-01565-5. 34480646

[101] Lim GB. Dyslipidaemia: PCSK9 inhibitor reduces need for lipoprotein apheresis in familial hypercholesterolaemia. Nat Rev Cardiol. 2016;13(11):634. doi: 10.1038/nrcardio.2016.148. 27629506

[102] Korman MJ, Retterstøl K, Kristiansen IS, et al. Are PCSK9 inhibitors cost effective? Pharmacoeconomics. 2018;36(9):1031–1041. doi: 10.1007/s40273-018-0671-0. 29777433

[103] Desai NR, Campbell C, Electricwala B, et al. Cost effectiveness of inclisiran in atherosclerotic cardiovascular patients with elevated low-density lipoprotein cholesterol despite statin use: a threshold analysis. Am J Cardiovasc Drugs. 2022;22(5):545–556. doi: 10.1007/s40256-022-00534-9. 35595929 PMC9468070

[104] Zhang Y, Chen H, Hong L, et al. Inclisiran: a new generation of lipid-lowering siRNA therapeutic. Front Pharmacol. 2023;14:1260921. doi: 10.3389/fphar.2023.1260921. 37900173 PMC10611522

